# Digital Government and Public Health

**Published:** 2004-09-15

**Authors:** Jane E. Fountain

**Affiliations:** National Center for Digital Government, John F. Kennedy School of Government, Harvard University

## Abstract

Digital government is typically defined as the production and delivery of information and services inside government and between government and the public using a range of information and communication technologies. Two types of government relationships with other entities are government-to-citizen and government-to-government relationships. Both offer opportunities and challenges. Assessment of a public health agency's readiness for digital government includes examination of technical, managerial, and political capabilities. Public health agencies are especially challenged by a lack of funding for technical infrastructure and expertise, by privacy and security issues, and by lack of Internet access for low-income and marginalized populations. Public health agencies understand the difficulties of working across agencies and levels of government, but the development of new, integrated e-programs will require more than technical change — it will require a profound change in paradigm.

## Definition of Digital Government

Digital government — also called e-government or virtual government — refers to governance affected by Internet use and other information technologies (IT). Digital government is typically defined as the production and delivery of information and services inside government and between government and the public using a range of information and communication technologies (1,2). The public includes individuals, interest groups, and organizations, including nonprofit, nongovernmental organizations, firms, and consortia. Because government functions exist at multiple levels in the United States, the impact of digital government varies widely across the country. The definition used here also includes e-democracy, that is, civic engagement and public deliberation using digital technologies.

The concepts of digital government that are relevant to organizational and institutional change arise from three fields of study: political science, organization theory (including social capital), and interactions of technology and organizational structure. The interplay among these ideas is highlighted in the uses of IT and the opportunity and challenges it presents.

Critical e-government topics include both societal and technical challenges and interactions between the two. On the *societal level*, the adaptation of government and civic engagement to increasingly computerized environments raises political, organizational, and social questions concerning use, context, reciprocal adaptation mechanisms, learning and the design of government work, the design of political and civic communities of interest, and the design of nation states in addition to international governance bodies (3).

On the technical level, IT is a tool, not a solution, but organizations rapidly absorb this sophisticated tool into everyday functions, so that IT becomes an essential part of the infrastructure of the organization. However, the most challenging role of IT is when it becomes a catalyst for change. If a government agency cannot effectively manage these changes, the organization may be overwhelmed (4).

## Historical Development

Initial efforts by government agencies to develop e-government entailed simply digitizing and posting static government information and forms on the Web using the language, displays, and design of existing paper-based documents. During the 1990s and continuing into the present, many government agencies have begun to adapt operations, work and business processes, and their interface with the public to simplify and integrate information and services in online environments.

The central governments of the United States, Canada, Finland, and Singapore are among those at the forefront of e-government in terms of the amount of information and interactivity available to the public and attention to system development and interface architecture. One of the key types of country-level initiatives is the country-level Web portal designed to help individuals navigate and search information for entire central governments. The U.S. government Web portal, www.FirstGov.gov, is an interface with a search tool meant to serve as a single point of entry to U.S. government information and services. The central government of Singapore developed a single Web portal, called Singov (www.gov.sg), to simplify access to government information for visitors, citizens, and businesses. Similarly, the Web portal for the Government of Canada, www.canada.gc.ca, was designed for three main constituents: Canadians, non-Canadians and Canadian businesses.

## Government Relationships

This section describes two types of government relationships with other entities. These are government-to-citizen (G2C) and government-to-government (G2G) relationships.

### Government-to-citizen

Digital G2C defines "citizens" as individuals and corporations. At present, digital government is most prized for its ability to improve communication with citizens (4-6). G2C exists in several forms, including agencies that offer e-based services, agencies that create a Web-based information site that allows searching and use of existing services, and virtual government portals that allow access to the services of multiple agencies. These efforts vary in interactivity and complexity. Maintaining a Web site increases outreach rapidly because the increased number of citizens on the Web increases the benefit to both government and citizens. In essence, G2C represents the first wave of governmental use of information technology: to provide information and services to citizens. It focuses on design and usability of Web sites but generally does not require extensive collaboration among government agencies. It emphasizes the first two roles of IT: tool and infrastructure.

Interactive e-government services include online tax payments, license applications and renewals, and grant applications and renewals. The City of Baltimore Web site (http://www.ci.baltimore.md.us/) has won awards for its implementation of computing technology in government. The system allows citizens to pay parking fines, property taxes, and water and other bills. Users can search crime statistics by geographic area within the city and track several city services, including trash removal and street cleaning. The City of Baltimore has implemented an online version of the 311 service available in some other large U.S. cities, which allows citizens to request city information and services over the telephone. Individuals can report and then track the status of a request for city services, including abandoned vehicle removal, pothole repair, graffiti removal, and requests for a change in traffic signs. These applications not only provide interactivity but also promote government compliance and accountability to voters by making provision of city services more transparent to the public.

Interactivity is increasing as governments continue to develop systems and citizens adapt to online government. For example, in the United States, the number of online federal tax filings increased from 20,000 in 1999 to 47 million, or about 36% of individual filings, in 2002. The Environmental Protection Agency reports that it saves approximately $5 million per year in printing and mailing costs by providing information digitally to the public.

### Government-to-government

Digital G2G delineates intergovernmental linkages. This is the second wave of IT use. Intergovernmental linkages are more challenging to implement because they require more integration within each governmental unit (4,7). G2G represents the third role of IT as a catalyst for change. G2G linkages may in the long run have the most radical impact on the function of digital government. Their establishment requires a much greater coordination within and between agencies and a movement away from oversight and budgeting processes that reinforce autonomous operations.

G2G development has lagged behind the activities of G2C because there is a less immediate payoff and more stress on each government agency. Government departments arise for a specific mission, often determined by law. These missions do not readily adapt to changing times, in part because of the oversight of legislative committees that are in turn affected by the advocacy groups with interest in the mission. Each of these groups has its own, often extensive, internal structures, and all these structures must align to allow major changes in agency interactions.

During the 1990s, several federal agencies and state-level governments created "virtual agencies," online sources of information and services from several agencies organized according to client group. For example, in the early 1990s, the U.S. federal government developed students.gov, seniors.gov, and business.gov to organize and display information using interfaces designed specifically for these populations with a single point of entry into a government portal. By the year 2000, there were approximately 30 cross-agency Web sites within the federal government.

Beginning in 2001, the development process shifted from a loose confederation of interested designers within the government to an enterprise approach to e-government, centrally managed and controlled, and used lead agencies to supervise projects. The desire for internal efficiencies drives these projects as much as concern for service to the public. Several payroll systems are being consolidated into a few payroll systems for the entire government. Multiple and abstruse requirements for finding and applying for government grants are being streamlined into one federal online grants system called e-grants. And myriad rulemaking processes in agencies throughout the federal government, while not consolidated, have been captured and organized in the interface architecture of one Web portal, called e-rulemaking. Recreation.gov uses an architecture that organizes recreation information from federal, state, and local governments. System design and interface architecture simplify search, navigation, and use of information on recreation activities, recreation areas, maps, trails, tourism sites, and weather reports by location. Standardization, consolidation, and integration of information, operations, and interfaces with the public have been the key drivers for e-government in most central government efforts.

## Challenges for Public Health

A major limitation to the effectiveness of digital government is the rigidity built into the structure of a bureaucratic state. This is why the accumulation of more sophisticated technology and specialists is insufficient to maximize digital government. Public health programs are well acquainted with the difficulties of working across agencies and levels of government. Health threats often arise from conditions that are outside the formal purview of the health department. An example might be a local industry that releases unhealthy pollutants into the community. In current bureaucratic systems, multiple organizations may have a role in addressing this problem: the public health department may detect a rise in pediatric asthma, the highway department may report more days of high-level pollution, and real estate companies may identify a drop in local housing prices. Bringing these systems together to take action requires leaders prepared to use new approaches. Public health leaders must look at the entire system to develop new, integrated programs. This is not a technical change but a profound change in paradigm.

Public health agencies are especially challenged by limited resources. Lack of funding for technical infrastructure and expertise means that the agency must be thoughtful about the technology it needs. IT offers many intriguing opportunities, but public health managers must identify the kinds of technology most critical to their mission. Managers, staff, customers, and IT specialists should be involved in such decision making. But how can programs assess their readiness for digital government?

- *Technical readiness *refers to both internal and external factors. Externally, are the agency's constituents able to access the Internet with sufficient skill and resources to benefit from the agency's Internet services? Internally, does the agency have sufficient infrastructure and skilled workers to support such services?
- *Managerial readiness* is independent of technical infrastructure. Does the agency have the organizational structure and culture to manage change? Do the managers as well as the IT specialists understand the potential impact of IT?
- *Political readiness* examines whether e-government is politically feasible. Will employees accept it? Will constituents? Will changes in the political arena affect support for e-government programs?

Many public health priorities are directed toward low-income and marginalized populations in which Internet use may be limited. Unequal access, roughly divided between those with education and those without, and highly correlated with income and political participation, maintains a digital divide in e-government despite advances in human-computer interaction (8,9). Lack of literacy and computer literacy exacerbates the digital divide. Disparities between rich and poor nations parallel digital divide challenges within countries. Yet innovations in several developing countries and in rural areas invite some degree of optimism. Rural farmers and crafts people are beginning to connect through the Internet to enhance their economic well-being. Rural communities in China are using the Internet, as yet on a modest scale, to decry local corruption and, in some cases, have forced the central government to intervene in local affairs.

Privacy and security concerns are issues for public health on several fronts. If an agency provides direct patient care, multiple regulations protect the confidentiality of medical records. If the vital statistics office is within the department of public health, state laws often indicate who has access to certificates and whether portions of certificates are confidential. The development of state and national systems linked to provide early alerts of potential environmental and biological terrorism raises issues of homeland security. These considerations are an essential aspect of IT programs and should be considered from the beginning of the design process.

## Conclusions

The technological potential exists for individuals, groups, and communities to participate in and shape government in new ways. Some observers speculate that increased access to government online will lead to greater interest, knowledge, and discussion of politics. The Internet might allow citizens to organize and mobilize resources in powerful new ways. Groups already civically engaged use computers to enhance their activities. However, the propensity to simplify and distort information in public discourse is not abated by changes in media.

Human-computer interaction begins with the study of the mutual adaptation of social and technical systems. It is not possible to predict the path or outcome of the many and varied complex adaptation processes now in play. One of the chief sources of learning for designers of e-government has been to focus on tools for building and sustaining democracy. While researchers learn more about human cognition, social interaction, and motivation within computer-mediated environments, and while designers are developing new tools and interfaces to encompass a wider range of activities and discourse within online environments, large-scale adaptation continues between societies, governments, and technology.

Deep-level changes in relationships among government agencies, the private sector, and nonprofit groups that maximize the opportunities of digital government require social capital. Social capital emphasizes mutual trust and support among entities and develops over years of interaction. While the term social capital is often used in discussions of geographic communities, it is equally important in virtual communities, and perhaps more so, because face-to-face encounters may be much less common in government relationships.

This social capital may offer the greatest benefit to public health as digital government moves forward. The synergy of different sectors working together can create innovations beyond the capacity of a single institution. While it may take several decades for government to maximize the adoption of G2G, agency interactions to promote good health for citizens is an essential element of public health. Public health leaders have a special responsibility to understand and expand the beneficial uses of digital government.
